# Initial experience with remote MRI scanning support in an oncology focused practice: Opportunities for expanded access to radiology care

**DOI:** 10.1002/acm2.70461

**Published:** 2026-01-07

**Authors:** Christopher M. Walker, Maria G. Maldonado, Megan C. Jacobsen, Suprateek Kundu, Michelle L. Underwood, Joshua P. Yung, Brandy J. Reed, David Jaffray, R. Jason Stafford, Marshall E. Hicks, Caroline Chung, Aradhana M. Venkatesan

**Affiliations:** ^1^ Department of Imaging Physics The University of Texas MD Anderson Cancer Center Houston Texas USA; ^2^ Department of Biostatistics The University of Texas MD Anderson Cancer Center Houston Texas USA; ^3^ Diagnostic Imaging The University of Texas MD Anderson Cancer Center Houston Texas USA; ^4^ Radiation Physics The University of Texas MD Anderson Cancer Center Houston Texas USA; ^5^ Interventional Radiology The University of Texas MD Anderson Cancer Center Houston Texas USA; ^6^ Radiation Oncology The University of Texas MD Anderson Cancer Center Houston Texas USA; ^7^ Department of Abdominal Imaging The University of Texas MD Anderson Cancer Center Houston Texas USA

**Keywords:** magnetic resonance imaging, practice expansion, remote scanning, technology, tool evaluation, workforce

## Abstract

**Background:**

As medical imaging demand grows, there is increasing stress on the currently available workforce to deliver consistent, high‐quality imaging studies while ensuring rapid study turnaround times and round‐the‐clock radiology coverage. Advances in remote access technology facilitating remote scan assistance and control are now commercially available to address these pressing clinical needs.

**Methods:**

This work evaluated an early clinical application of a virtual scanner operations system (syngo Virtual Cockpit (VA13A, Siemens Healthineers, Erlangen, Germany) for remote magnetic resonance imaging (MRI) monitoring and scan control at three geographically distant outpatient sites associated with our primary institution.

**Results:**

The system facilitated execution of technically complex oncologic MRI exams at these geographically distant clinics with no measurable impact on acquisition time compared to MR imaging performed at our primary hospital location. Additional operational improvements were realized with the use of the system, including remote staff training, technical assistance, and scanning during staff shortages. This early iteration of remote scanning had some limitations including limited utility for additional assistance in the scanning of those protocols that require complex physical setup. Moreover, connectivity issues were noted to be a limiting factor that contributed to operational delays. It was still necessary to have an onsite MRI technologist at the scanner console to interface with the patient and ensure safe operation.

**Conclusion:**

Despite these limitations, our initial experience demonstrates that the use of remote MRI scanning support facilitates staffing flexibility while providing expanded patient access to oncology MRI services.

## 1 INTRODUCTION

Increasing global investments in healthcare and technological advances in image quality and acquisition speed have resulted in a dramatic increase in the worldwide accessibility and use of medical imaging.^1^, ^2^, ^3^ As a result, diagnostic radiology practices are growing around the world, both in terms of the number and complexity of clinical studies ordered.^1^ To accommodate the increase in acquisition complexity, many large radiology practices employ teams of specialized technologists with expertise in scanning advanced clinical and research protocols,^4^ as well as in‐house imaging physicists, to assist with uniquely challenging cases. This organizational structure can lead to operational restrictions on protocol availability that may limit access to care at distant outpatient centers and lead to additional patient travel time, increased cost, and lower satisfaction.^5^, ^6^, ^7^, ^8^

Adding to this complexity is the growing number of contemporary radiology practices providing care across a wider geographic network, which necessitates adequate numbers of trained imaging technologists and supervisory staff at each site. These factors represent significant challenges to the provision of consistent, high‐quality imaging care, particularly across large areas, given finite personnel resources.^9^ This challenge, already a reality for many radiology groups and departments, was particularly exacerbated by the COVID‐19 pandemic and remains an ongoing challenge for many in the post‐pandemic setting.^10^ Lack of access to specialized personnel is particularly acute in marginalized or low‐resource areas.^11^, ^12^ This can result in reduced quality or availability of radiology care.^13^

Remote access technologies can enable provision of remote imaging expertise during scan acquisitions to address these system complexities and circumvent the problem of finite personnel resources. Remote technologies are widely used for diagnostic radiologic interpretation, with interpretations commonly performed by radiologists at remote workstation.^14^ A similar concept has been implemented by many vendors (Siemens Healthineers, Erlangen, Germany; GE HealthCare, Waukesha, WI; and Philips, Amsterdam, Netherlands) that allows for both remote scan assistance during image acquisition as well as remote scan control/operation.^15^ Types of remote access technologies that exist include vendor‐neutral platforms that can integrate with scanner hardware to enable remote scan monitoring and control as well as a range of two‐way audio and visual communication platforms permitting communication between monitoring sites and the distant geographic sites. While the field of remote scanning is rapidly developing, a range of remote interventions are already permissible with use of these technologies, ranging from brief remote assistance from a radiologist to protocol parameter optimization, scan troubleshooting from a physicist, or complete remote scan operation by a skilled technologist located at a distance from the scanner site. This emerging technology presents opportunities^16^ and risks.^17^ A critical consideration is to ensure remote scanning does not diminish patient safety by reducing staffing beyond recommended guidelines. A detailed discussion of these considerations can be found in the American College of Radiology Manual on MR Safety and related recent reviews.^18^, ^19^

We evaluated the operational impact of one of the earliest virtual operation systems approved for remote scan monitoring and scan control of MRIs located at three outpatient facilities geographically distance from our primary institution. This work summarizes our initial experience with the use of this system and its ability to facilitate advanced imaging of complex oncologic MRI protocols executed at remote sites.

## 2 METHODS

### 2.1 Remote scan operation software overview

The Syngo Virtual Cockpit (VC) (Siemens Healthineers, Erlangen, Germany) is a remote assistance tool that allows remote users to interface with an MRI system, permitting full access to the scan console and featuring scan operation capabilities. The VC system used in this study (VA13A) was released for clinical use in 2021 included two‐way communication interfaces with the technologist physically at the scan console via audio, visual, and text‐based communication. There was a patient‐facing camera for remote monitoring of the patient. An additional camera provided a real‐time video feed of the power injector console at the remote scanner. The VC system had many modes of operation and data flow. Screen sharing and virtual system input were integral aspects of the system, which allowed a remote user to see the scanner interface as well as interact with it if necessary. Communication between the assistant at the main hospital command center and the technologist at the remote scan console was facilitated by either a chat interface or built in voice over IP communication. There was additional real‐time video feed of the patient bed and the power injector console for the remote assistance to monitor, though the remote assistant was unable to interact directly with the power injector or communicate directly with the patient. These tasks were performed by the technologist at the remote scanner console.

**FIGURE 1:**
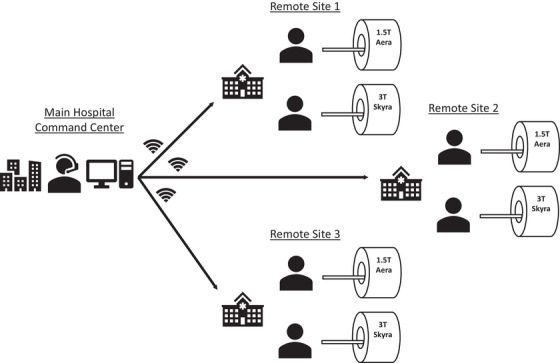
Schematic layout of the remote MRI scanning system (syngo Virtual Cockpit, Siemens Healthineers, Erlangen, Germany). The main hospital command center was the location where the senior MRI technologist operated, providing remote scan support or remote scan control over the three geographically distant locations for a total of six MRIs.

Our institution's installation of the VC system included a VC command center at our main hospital location, with communication capabilities between the command center and each of three distant outpatient imaging clinics, as shown in Figure 1. The main hospital command center comprised a workstation equipped with audio‐video capabilities that was installed near the offices of an advanced technologist team. Monitoring cameras and additional software to enable VC console access were installed on the remote MRI scanners. Four days of onsite training was provided by the vendor for the technologist team using the VC system. In person training was provided to both the main hospital technologist team as well as the technologist at the distant outpatient imaging clinics.

### 2.2 Virtual cockpit patient population

**FIGURE 2:**
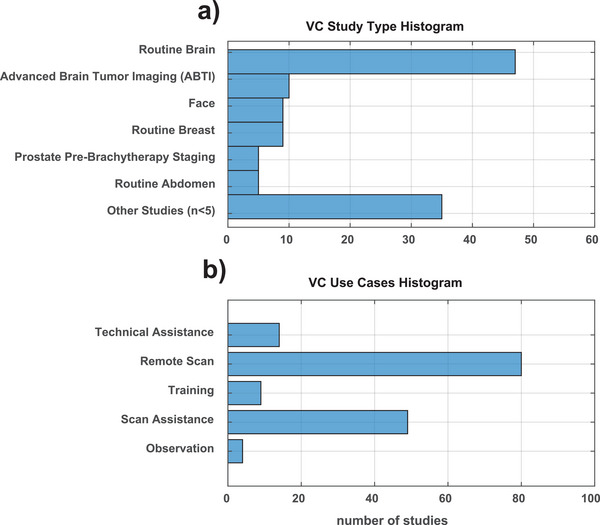
Bar graph summarizing the numbers of oncology MRI studies acquired with the use of a remote assistance platform versus no remote monitoring. Primary usage was for routine brain MR imaging with some utilization for routine breast, face, and abdominal MRIs as well as advanced studies like our advanced brain tumor imaging (ABTI) protocol or prostate pre‐brachytherapy staging and workup MRI.

The VC system was evaluated from March 18, 2021 until September 2nd, 2022 and all studies acquired with remote assistance were recorded. All scanning was performed on either a 1.5T Magnetom Aera or a 3T Magnetom Skyra system (Software Version VE 11; Siemens Healthineers, Erlangen, Germany). Studies acquired are outlined in Figure 2 and ranged from a routine breast MRI protocol to advanced brain tumor imaging (ABTI) protocol, which includes multiple quantitative and semi‐quantitative MRI sequences, such as dynamic contrast‐enhanced (DCE), dynamic susceptibility contrast (DSC), and spectroscopy. Advanced studies were defined as any study whose complexity required acquisition by a senior MRI technologist, such as dedicated breast MRIs, and/or studies not routinely performed at our remote site locations, for example, our prostate pre‐brachytherapy staging and workup MRI. The primary users of the VC platform were MRI technologists at the remote scanner consoles who received assistance largely from a specialized team of senior technologists who were staffing the main hospital command center. Use of the VC to facilitate scanning ranged from monitoring and audio assistance to fully remote scan control and study acquisitions as outlined in Figure 2b. Remote scan assistance also included other activities, such as remote technologist training and education, protocol management, and technical assistance.

### 2.3 Operational performance analysis

Operational performance metrics were calculated from study DICOM headers and included derived metrics such as table time. Table time was defined as the time between the initiation of the localizer and the completion of the final imaging series in the study. The operational metrics for these studies were compared to a control group of brain (*n* = 276) and breast (*n* = 58) MRIs performed without VC assistance at our main campus location during the same time period. For this non‐inferiority analysis, we consider less than 5 min for simple studies and less than 10 min for complicated studies to be an operationally insignificant change (Table S1). Technical issues that caused scan delays, limited communication, or connectivity errors were tracked to determine the most frequent failure modes for a VC assisted procedure.

### 2.4 Case studies

Three protocols were utilized as case studies to demonstrate the utility of the VC. The first evaluated the efficacy of training technologist staff at remote sites to acquire a DSC series as part of the routine Brain MRI protocol. The second case study demonstrated how specialized staff could scan ABTI exams remotely with the VC, and the third evaluated the expansion of the prostate brachytherapy simulation program to remote sites.

## 3 RESULTS

### 3.1 Patient population

Virtual cockpit assistance was utilized for 120 studies, with 24 of those studies classified as advanced or non‐routine studies. The types of MRI studies scanned with VC assistance are summarized in Table 1. A histogram demonstrating the frequency of the studies performed with each protocol and type of remote assistance is presented in Figure 2. The most common studies performed with VC remote assistance were routine brain MRIs and routine bilateral breast MRIs.

**TABLE 1:** Summary of studies performed with remote assistance from Virtual Cockpit (VC) as well as reference studies scanned as a part of routine operations.

Summary of studies performed with remote assistance using the virtual cockpit					
Study description	Number performed with VC	Number of reference studies	VC scan time (min.)	Reference scan time (min.)	Feasibility without VC support
**Routine brain**	47	276	30.9	29.5	Yes
**Routine breast**	9	58	38.5	35.7	Yes
**Advanced brain tumor imaging**	10	54	53.3	50.1	No
**Face**	9	–	–	–	Yes
**Prostate pre‐brachytherapy**	5	–	–	–	No
**Routine abdomen**	5	–	–	–	Yes
**Other studies (** *n* ** < 5)** ^*^	35	–	–	–	Yes

Note: * MRI studies performed with VC support infrequently (*n* < 5) included, but were not limited to, routine abdomen, spine, femur, inner auditory canal, skull base, and liver elastography. Column six indicates if it was possible to perform the protocol at the geographically distance site without VC support.

### 3.2 Operational performance analysis

**FIGURE 3:**
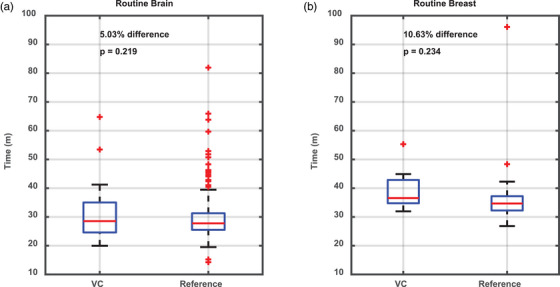
Comparison of table times for routine brain (a) and breast (b) MRIs scanned with the use of the VC system (VC) versus conventional scanning performed at the main hospital command center during the same time period. Table times commenced at the start of the acquisition of the localizer sequence and concluded after acquisition of the last series.

The operational metrics for routine brain and bilateral breast studies were compared to a control group of brain and breast MRIs performed without VC assistance at our main campus location. As seen in Figure 3a, routine brain MRI scan times were marginally increased from 29.5 to 31.0 min, an increase of 5.0%, (*p* = 0.219). Similarly, breast MRI exam times were also marginally increased from 35.7 min to 39.5 or 10.6% (*p* = 0.23). While these initial adoption results cannot demonstrate an absence of increased scan time with the utilization of the VC, they do suggest that the increased scan time was not clinically significant.

**FIGURE 4:**
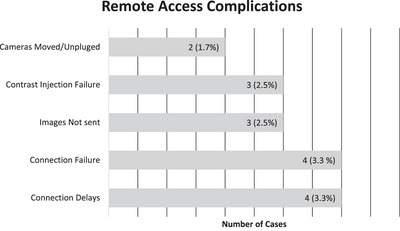
Summary of failure modes and frequencies. Bar chart summarizing causes for delay or termination of a study while using the remote assistance platform.

The additional technical complexity required for remote scan assistance and scan control is not without its limitations. There were delays associated with the remote scanning platform as outlined in Figure 4, with a frequency of ∼3.33% (4 out of the 120 studies). The most common causes for delay were connectivity issues which either resulted in delayed assistance or no assistance if connectivity could not be established between the main hospital command center and the three geographically distant MRI sites. Failure to correctly utilize the cameras at the remote sites was also a source of delay when cameras were moved or unplugged at the three remote MRI sites, contributing to delays in the execution of remote scan monitoring until appropriate camera connectivity and positioning was established. Other limitations were encountered when network related delays between the main hospital command center and the remote site impeded the image feed of the power injector, limiting the ability to accurately monitor contrast injections and the timing between injections and series acquisitions when performing remote scans. If the network related time delay of the injector camera feed is known to the scanning technologist, scan initiation can be compensated to ensure proper acquisition times of dynamic phases for that particular study.

While the primary responsibility of the onsite technologist was patient safety, which they were ultimately responsible for, scanning assistance by the technologists at our main hospital command center allowed for the MRI technologists at the remote geographic locations to perform other clinical activities, while still being readily available to assist with any patient management or safety‐related occurrences. Common activities the onsite technologist performed during remote MRI scanning included reviewing and updating the clinical schedule, preparing for upcoming cases, and participating in training or on‐site staff meetings.

## 4 CASE STUDIES

### 4.1 Dynamic susceptibility contrast sequence training

**FIGURE 5:**
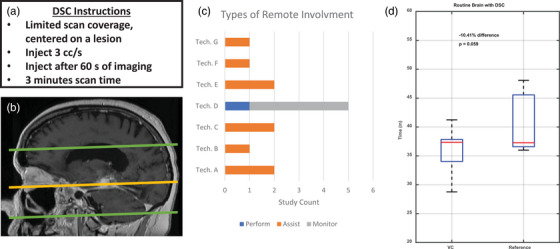
Implementation of a new dynamic susceptibility contrast (DSC) sequence for brain tumor imaging. (a) Multi‐step technologist instructions and (b) defined plane prescriptions are necessary when a DSC sequence is added to the routine brain study. (c) Bar graph summarizing the types of assistance leveraged by onsite MRI technologists at our remote geographic locations using the remote MRI assistance platform. (d) Comparison of table times for routine brain MRI studies acquiring the additional DSC sequence with the use of the virtual assistance platform (VC) compared to conventional cases acquired at the main hospital during the same time period.

The Virtual Cockpit facilitated training at our remote sites, such that technologists at these locations were educated in the scanning of new sequences and protocols. Figure 5 illustrates the process used for protocol dissemination, the types of assistance leveraged using remote assistance, and a comparison of scan times at the various levels of remote interaction. Most technologists required only one or two supervisory sessions via remote scan monitoring before they were able to perform the additional series independently. In some instances, the senior technologist at the main hospital command center performed the entire acquisition, (See Figure 5c, Technologist D). These types of remote scanning assistance resulted in a slight, but not statistically significant, reduction in scan time of 10.4% (36.3 min compared to 40.6 min, *p* = 0.06) compared to the same protocol change being run by technologists scanning at the main campus site using conventional technique, i.e., without remote assistance.

### 4.2 Advanced brain tumor imaging protocol

**FIGURE 6:**
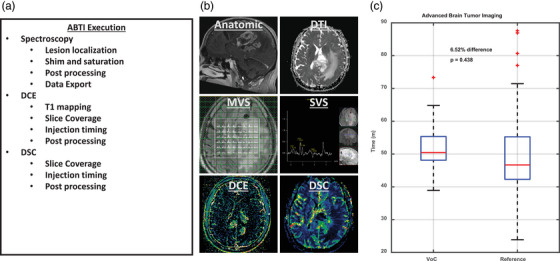
Operational steps for successful scanning of advanced brain tumor imaging (ABTI) MRI protocol and scan times with and without VC assistance. ABTI studies include multiple quantitative or semi‐quantitative MRI scans, including DCE, DSC, and spectroscopy. These advanced scans necessitate expertise in technical execution and are typically performed by a specialized team of senior technologists. To ensure high‐quality DSC scans, slices must be carefully centered on the brain mass, with a limited slice range. Proper injection rate and scan timing are essential. Spectroscopy must also be performed, with a focus on voxel location and B0 shimming centered on the lesion. In addition to technical expertise, extensive post‐processing is required for these acquisitions in order to produce quantitative imaging biomarkers like k‐trans or relative cerebral blood volume. To ensure consistency, post‐processing is performed on the scanner console before the images are pushed to PACS. (a) Summary of the necessary steps required from the senior technologists to perform the advanced tumor brain imaging MRI protocol. (b) Representative examples of single slice axial brain MR image outputs. (c) Comparison of table times for studies performed with the use of the remote assistance platform by a senior MRI technologist at the main hospital command center compared to the studies performed at the main hospital by the same group of senior technologists.

The use of the VC also enabled our senior MRI technologists at the main hospital command center to scan complex oncologic MRI protocols that had not been scanned before at the geographically distant MRI sites due to a lack of trained on‐site staff. These studies included our advanced Brain Tumor Imaging (ABTI) protocol. The technical steps necessary for successful execution of the ABTI protocol are outlined in Figure 6a and b. Using the remote scanning platform, thirteen ABTI studies were acquired. All ABTI studies were acquired without significant increases in acquisition time compared to reference studies acquired on the main campus (53.3 min vs. 50.1 min *p* = 0.44, Figure 5) again suggesting that a non‐significant < 10% increase in table time can be expected with remote scan operation.

### 4.3 Prostate brachytherapy simulation for treatment planning

**FIGURE 7:**
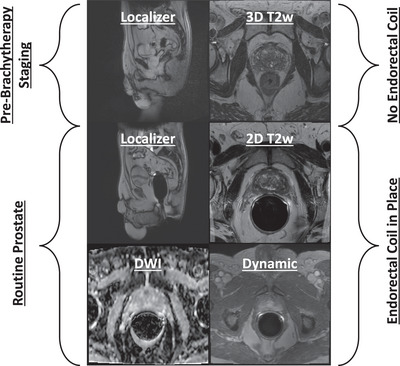
Schematic and representative images of the prostate pre‐brachytherapy staging MRI protocol. Compared to the routine prostate MRI protocol, one additional 3D T2‐weighted sequence is acquired before the insertion of the endorectal coil, which is employed for brachytherapy treatment planning.

Dedicated MRI protocols performed for prostate brachytherapy simulation are also scanned by a specialized team of senior MRI technologists at our institution and therefore had not previously been scanned at the geographically distant locations. This protocol is similar to a staging prostate MRI performed with an endorectal coil but includes an axial 3D T2‐weighted sequence of the prostate employed for radiotherapy treatment planning, which is acquired prior to the insertion of an endorectal coil. An overview of the protocol is shown in Figure 7. The axial 3D T2 sequence requires additional coverage and anatomical landmarks to identify the potentially dose limiting organs‐at‐risk for treatment planning. Failure to properly cover the needed anatomy can limit treatment planning, such that a repeat study is required. For these studies, our senior technologist at the main hospital command center remotely acquired the approximately 7‐min 3D T2 treatment planning sequence, and then the onsite technologist at the remote site completed the remaining routine prostate series.

## 5 DISCUSSION

The ability to remotely monitor and control an MRI scanner has significant potential to improve workflow efficiency, facilitate technologist training and education, and maintain clinical productivity despite finite numbers of trained personnel and/or staff shortages. We report our preliminary experience using a remote MRI scanning support system permitting remote monitoring and scanning of complex oncologic MRI studies. We observed that many technically advanced studies could be performed remotely, expanding access to these studies for our patients across a broader geographic network than had previously existed. We also observed that having the flexibility of a remote operator who was able to take over some or all of a study's acquisition, allowed the onsite technologists at our remote geographic sites the ability to address additional clinical and administrative responsibilities, while still being available to ensure patient safety. Patient safety is best managed by trained and experience onsite personnel.^17^ For this study the MRI staffing model was not modified to account for the inclusion of remote scanning, and the staffing was kept at or above levels recommended by the American College of Radiology.^18^, ^19^ This work used the remote technologist exclusively as an adjunct to the scanning operations yet still found opportunities to improve operations. The inclusion of a second technologist in the scanning process might not seem like an obvious strategy for improved efficiency. However, MRI technologists have a multifaceted role in the delivery of MRI related care, and allowing flexibility regarding which person is actually acquiring the data allows for management of other aspects of MRI operations that could otherwise delay the practice.

In our experience, the use of this remote scanning support system improved training efficiency for MRI technologists at remote geographic sites. Incorporating new sequences into routine protocols is essential to ensure the delivery of the most up‐to‐date imaging studies. However, these updates may impact operational efficiency as the scanning technologist learns and adjusts to the new or updated protocol. This transition period represents an opportunity for virtual tools to assist in training and implementation, reducing the effort needed to maintain a state‐of‐the‐art imaging practice. As an example of this, a derivative protocol of our routine brain was implemented that includes a dynamic susceptibility contrast‐enhanced (DSC) series acquired before the routine post‐contrast T1‐weighted images for lesion characterization. DSC acquisitions require precise injection and acquisition timing and require different slice selection than other portions of the routine brain study due to coverage limitations. The VC was used to assist and train the technologists at our remote sites on the execution of this new sequence. The tool's flexibility allowed for varying levels of remote interaction, from non‐invasive observation of the onsite technologist performing the sequence to near complete remote scan execution by the senior technologist at the main hospital command center.

There are multiple additional roles for remote scanning systems that we utilized but which were not fully explored in this work. Protocol management in MRI is challenging, especially for geographically disparate scanners. While tools are being developed to help manage fleet wide protocols, they are still developing and have their own set of limitations depending on the platform used. The ability to remotely make protocol modifications can alleviate the need to travel to remote sites or to have to explain protocol changes over the phone or via email which can be compromised by communication errors. While offering several advantages, the remote scanning support system evaluated by this work necessitated an operator present at the scan console to initiate the remote connection, even for activities such as protocol management, which ideally could be performed when the clinic is closed. Remote assistance can also be provided by a radiologist, physicist, or trained Magnetic Resonance Safety Expert to assist with a challenging case such as the imaging of patients with medical implants that require real‐time protocol modifications to ensure device‐specified MRI conditions are not exceeded. These experts can also help troubleshoot image quality issues as they occur and help optimize a study's image quality before it is completed. Remote scan monitoring and assistance can also be used for routine testing of the system when an onsite technologist is present to set up any phantom studies which would then be run and analyzed by a remote physicist, again reducing senior staff travel burden to the remote location. All of these use cases were employed during our evaluation period to a limited extent and all demonstrated potential for additional clinical impact.

There were some limitations with remote scanning encountered in this study. Direct patient contact activities still require on‐site trained personnel for patient management, though this may change with technology improvements or regulatory changes. As practice evolves it is important to remember how reliant MRI safety is on the onsite MRI technologist. While patient facing cameras were available, their view through the scan room window was limited, necessitating that proper patient or phantom setup is performed by knowledgeable onsite personnel. This is essential for the setup of critical procedures such as MR elastography studies or functional MR imaging. Communication is an additional limitation encountered, as the senior technologist at the main command center could only audibly communicate to the onsite technologist at the remote scanner console, and could not communicate within the scan room; the ability to communicate while assisting a patient on the table was limited. There were other practical limitations associated with any information technology, including connectivity delays or failures which could degrade, delay or even terminate patient care, depending on the extent and criticality of the remote assistance. All of these factors should be considered by sites as they explore the use of remote scanner operations to ensure the operational needs are met by the system. Additionally, the network infrastructure needs to be compared to system specifications to ensure seamless operation. This highly technical and clinical integration is an opportunity for consultation with members of the medical physics community.

Remote access technologies are being increasingly adopted in the healthcare setting, making computing environments independent of physical infrastructure and staffing constraints. This can improve clinical efficiency, operational flexibility, and expand access to care. This work summarizes our initial experience with a virtual scan monitoring platform permitting remote monitoring and remote scan control. We found that virtual scanning was able to enhance clinical operations, improve operational flexibility, and expand access to complex and advanced oncology MRI protocols to our patients across a broad geographic network.

## AUTHOR CONTRIBUTION

*Concept and design*: Aradhana M. Venkatesan and Caroline Chung. *Acquisition, analysis, or interpretation of data*: Christopher M. Walker, Suprateek Kundu, and Maria G. Maldonado. *Drafting of the manuscript*: Christopher M. Walker, and Megan C. Jacobsen. *Critical review of the manuscript for important intellectual content*: All authors

## CONFLICT OF INTEREST STATEMENT

This work was supported by Siemens Healthineers (Erlangen, Germany) and by the University of Texas MD Anderson Cancer Center‐Siemens Alliance.

## Supporting information



Supporting Information

## Data Availability

The data that support the findings of this study are available from the corresponding author upon reasonable request.
