# Supplementary material for: Initial experience with remote MRI scanning support in an oncology focused practice: Opportunities for expanded access to radiology care
Source: J Appl Clin Med Phys. 2026 Jan 7;27(1):e70461. doi: 10.1002/acm2.70461 (PMC12779931; doi:10.1002/acm2.70461)
Supplement: Supplementary file 1 — Supporting Information [file ACM2-27-e70461-s001.docx]

**Supplemental Information**

| **Study** | **Ref. Mean** | **Ref. Std.** | | **Ref. Count** | **VoC Mean** | **VoC Std.** | **VoC Count** | **Inferiority Margin** | **Alpha** | **Power (1-β)** |
| --- | --- | --- | --- | --- | --- | --- | --- | --- | --- | --- |
| **Brain** | 29.48 | 7.48 | 276 | | 30.96 | 8.44 | 47 | 5 | 0.25 | 0.75 |
| **Breast** | 35.72 | 9.01 | 58 | | 39.52 | 7.33 | 9 | 10 | 0.25 | 0.7 |
| **ABTI** | 50.07 | 12.52 | 54 | | 53.33 | 9.67 | 10 | 10 | 0.25 | 0.58 |
| **DSC** | 40.57 | 5.48 | 5 | | 36.35 | 3.42 | 14 | 5 | - | - |

Supplemental Table 1: Statistical description of the scan time analysis. All times are in units of minutes.
